# Supplementary material for: Dual-light photodynamic therapy administered daily provides a sustained antibacterial effect on biofilm and prevents Streptococcus mutans adaptation
Source: PLoS One. 2020 May 6;15(5):e0232775. doi: 10.1371/journal.pone.0232775 (PMC7202659; doi:10.1371/journal.pone.0232775)
Supplement: Attachment — Submitted filename: Response to Reviewers2.docx [file pone.0232775.s002.docx]

PLOS ONE

Comments for editor and the reviewers:

Revision of the manuscript: ‘Dual-light photodynamic therapy administered daily provides a sustained antibacterial effect on biofilm and prevents *Streptococcus mutans* adaptation’

We kindly thank the editor and the reviewers for their time and efforts to revise our manuscript. We found the advice and thoughts very relevant and are very glad for the help provided.

**Reviewer #2**

1. **The revised version is now improved. All points and suggestions of the reviewer are fulfilled. The revised version can be now recommended for publication.**

We kindly thank you for your time and efforts to improve our publication.

**Reviewer #3:**

**An interesting manuscript looking at the potentiated antimicrobial effects using a combination of PDT and aBL against S. mutans biofilm. Before acceptance of the manuscript, the following points should be addressed.**

We kindly thank you for your interest towards our work and we greatly appreciate your help in improving our paper. The clear presentation of these comments helped us greatly to address the issues.

1. **Line 97 – the citations only seems to include resistance to PDT and not aBL, should add some aBL citations.**

A: We added the following citations, see line 97:

Guffey SJ, Payne W, Jones T, and Martin K. Evidence of Resistance Development by Staphylococcus aureus to an In Vitro, Multiple Stage Application of 405 nm Light from a Supraluminous Diode Array Photomedicine and Laser Surgery. 2013, Vol. 31, No. 4

Yucheng Wang, Ying Wang,a,Yuguang Wang,a,e Clinton K. Murray,f, Michael R. Hamblin, David C. Hooper, and Tianhong Dai. Antimicrobial blue light inactivation of pathogenic microbes: state of the art [Drug Resist Updat. 2017 Nov; 33-35: 1–22.](https://www.ncbi.nlm.nih.gov/entrez/eutils/elink.fcgi?dbfrom=pubmed&retmode=ref&cmd=prlinks&id=29145971)

2. **Line 106 –were the replicates performed independently on 6 different days?**

We have performed all the experiments of a given study protocol during the same day, simultaneously. This enabled us to use the same *S. mutans* suspension, identical light exposure parameters and provided exactly similar laboratory conditions. We believe that the samples to be comparable with each other, the conditions should be kept as similar as possible.

Added in the text line 109: All the replicants in each study protocol were performed during the same day, simultaneously. This enabled to use the same *S. mutans* suspension, identical light exposure parameters, and provided exactly similar laboratory conditions.

3. **Line 140 –it would be nice to have the absorption spectrum of the photosensitizer**

A: Absorption spectrum of ICG can be seen under the title: ‘Absorption spectroscopy analysis of ICG adherence to *Streptococcus mutans’* and seen in Fig 6.

Added in the text, line 142: Absorption spectrum of ICG is provided below.

4. **Line 152 – add emission spectra of all light sources used**

A: We provided Table 2 to provide this information.

Added in the text, line 153: The emission spectra of the used light sources are presented in Table 2.

5. **Line 154 – should be written 100 mW/cm2 instead of 100mW/cm2 – this should be changed for all within the manuscript.**

A: This change has been made. We did the same change from 100J/cm2 to 100 J/cm^2^ as well.

6. **Line 164 – what is meant by ‘relative amounts?’ the authors need to be very clear**

A: Good point, thank you. This definitely clarifies the message. We went through the paper to clear this problem.

Added in the text, line 169, among other changes: **We also tested the antibacterial efficacy of dual-light treatment in terms of different relative amounts of 810-nm and 405-nm light irradiance, when the total amount of light was kept constant.**

7. **Line 173 -what was the purpose of adjusting the irradiances using these ratios? was it to ensure similar durations of exposure? Was duration of the treatment most important to the authors? I would have thought distributing ratio’s in terms of radiant exposure may have made more sense.**

A: This is a good point. The amount of radiant exposure (J/m^2^) was the constant of interest, and the changing irradiance (W/m^2^) ratios of aBL and aPDT light sources reflect to that. The text just doesn’t follow this idea clearly enough. We have carefully revised the manuscript, clearing the idea of radiant exposure being the changing ratio.

Changes in the text have been revised throughout, see especially paragraph starting at line 169.

8. **Line 239 - 0 colonies on the plate does not mean 0 CFU, you are limited by the threshold for detection based on the dilution factor. Please amend your figures to factor in the limit of detection.**

According to the observed biofilm mass in the bottom of the well in each experiment, the dilutions were performed accordingly. As an example, treated biofilms were most usually serially diluted to from 1:1 to 1:10^4 and controls usually 10^5-10^6, with a single plating from each dilution.

The CFU 0 results were obtained with 1:1 dilution factor. Biofilm from well was mechanically removed to 200ul of buffer from which 100ul was plated to agar. If bacteria were viable, they should form a countable colony to agar in two days. This means that in these l zero results there were no viable bacteria in plated 100ul of the sample. All experiments were done with identical biofilm extraction procedure and in 6 repeats to mitigate small statistical errors in sampling. The sampling errors are small compared to logarithmic differences between different treatments.

Added in the text, line 190: The CFU 0 results were obtained with 1:1 dilution factor

9. **Line 308 – again please be more specific than ‘relative amounts’**.

A: Thank you for these comments, helping us to communicate our results more clearly.

We have thoroughly revised the manuscript regarding ‘relative amounts’, irradiance and radiant exposure. Please see the Manuscript track changes-version, line 53, line 171, line 315, line 331, line 398 and line 470.
